# Modes of Practice and Their Vicissitudes

**Published:** 1860-05

**Authors:** J. Taft


### MODES OF PRACTICE AND THEIR VICISSITUDES.

BY J. TAFT.

In taking a retrospect of dental practice for the last ten or fifteen years, there are many particulars, both in the mechanical and operative departments, to which it may be interesting, if not instructive, to advert occasionally, to modes of practice that have been introduced, and the ordeals to which they have been subjected. We will be content for the present, to consider a little some of the methods and styles of work in the department of artificial dentures, or rather, in the mechanical department. The first method introduced for obtaining artificial dentures was by carving from ivory or bone, or the teeth of some animal, the substitutes, or using the human teeth. These were usually carved, or formed to fit the parts as nearly as the eye would direct. They were sometimes inserted upon metal, in other cases, without metal; if a partial set, with clasps or ligatures; if an entire set, by springs, never by atmospheric pressure. This method of procuring artificial teeth is wholly abandoned, because it is superseded by others much superior.

The next step in the advancement was the production of porcelain teeth, and these were mounted upon gold or silver plate. In the introduction of metallic plates, they were adapted to the parts as nearly as possible with the pliers and hammers, without models. We are not advised as to the time when forming models from impressions taken from the parts, was introduced ; it was probably thirty years ago. This process revolutionized the adaptation of plates to the gums. In the production of these, many improvements have been made from time to time ; they were first made of brass, copper, and sometimes of iron, but these were found impracticable, not only from the difficulty of procuring them, but also because of the great contraction to which these metals were subject, and other metals have been introduced, that have sufficient hardness, and will contract but little or none at all, that will run sufficiently sharp to give a perfect model of the part represented. This process will doubtless be retained as long as bases are made for artificial teeth of wrought metal.

The method of mounting teeth upon gold or silver plates was far more successful in the production of artificial dentures than any thing that had preceded it. Fifteen or twenty years ago, teeth well mounted upon gold or silver plate, was supposed to be the ultimatum of mechanical dentistry ; long since that, and some even to the present time, seem to rest quite satisfied with only this attainment in the production of artificial denture; but the improvements that have been made in the last ten years have taken away much of the prestige of this style of work.

Continuous Gum Work was introduced to the profession about ten years ago, and though very crude and imperfect at first, it has been improved step by step, till it is now conceded to be in many particulars superior to every thing else. It at the out-start promised much, and owing to this fact, many were induced to take hold of it, and many of these having no ability to use it, even had it been in a greater state of perfection. Efficiency in the early part of its history, even in the hands of those most skillful in its use, was the exception, and not the rule. The consequence was, that a large proportion of those who first took hold of it, sooner or later abandoned it, either to hold forever in remembrance their sad disappoinment, wtithout it, or to resume it when more nearly perfected, and under more encouraging auspices. This style of work has run a severe course from the beginning; it involves a process and manipulation requiring great skill, tact, and rather an extensive knowledge, in certain directions. This fact, the greater proportion did not recognize, for a time at least. Under such circumstances as these it was, that this style of work received such fearful denunciations. The successful production of this work involves some principles of science, not very generally attained by the dental profession. The precise influence of heat upon metals and upon silicious bodies is not yet very perfectly comprehended, even by those farthest initiated, but carefully conducted experiments are leading in the proper direction.

He who sets at defiance principles well established, either by experiment or otherwise, should not complain if he meets with unfortunate results. It would be tedious to designate all the mistakes that have been made in the construction of this work; suffice it to say, that from the beginning to the end, there is scarcely a step where errors have not been made—in working the platinum, annealing it, swaging it, attaching the teeth, in the preparation of body and gum, in baking it, in the management of the furnace, etc.—all these require definite and accurate manipulation, based upon correct principles. The value and efficiency of this work is no longer problematical. There are, however, without doubt, improvements to be made upon it; perfection we have not anywhere. Continuous gum work has run a severe gauntlet, but is coming out a success.

Cheoplastic, or Cast Work.—This style of work was presented to the profession between three and four years ago. With the method of its presentation all are familiar. Many advantages were claimed for the work, the chief of which is the perfection of adaptation; the base being cast into the plaster model, and running sharply, and at a low temperature, and contracting but little or none in cooling, the adaptation is necessarily very accurate. The metal may be worn in almost all mouths without any very objectionable change.

The entire obliteration of all interstices by this mode is a very desirable feature. Any desired form, prominence or thickness can be given to this work. This style of work was adopted at first by a large number in the profession, quite a number of whom have abandoned it. Some there were whose expectations were almost wholly disappointed, because they expected too much, anticipating that it would supplant and take the place of every other style of work, and that the work would be accomplished with far less labor than by any other mode.

Some persons in the dental profession, as everywhere else, eagerly grasp at, and become enthusiastic over anything and everything that is new, regardless of its real merits. Others again, with eagerness take hold of anything that promises profit; others there are—lazy souls—who readily adopt any mode of practice that can be easily and quickly accomplished. Again, there are some who examine carefully, in the light of science, the real merit of any mode of practice, and then testing it by careful experiment, determine accurately the true value of the process, and then adopt or reject, according to this umpire. Cast work of pure tin was introduced to the notice of the profession some ten or twelve years ago, but was not very extensively used, owing probably to its contraction upon cooling, and a want of sharpness in casting. Cheoplastic method may be employed in the construction of appliances for the correction of irregularity. It is in this particular valuable. This style of mounting teeth, like all others, has some objections, the chief of which are its weight and the character of the metal; and this is generally most objectionable to those who know least about it. It can not with impunity be worn in the mouth with gold or platinum plates.

Vulcanite Work.—This style of work was introduced to the profession about five years ago. It has gradually been gaining favor with the profession from the beginning. The method of its introduction was not such as would make the best impression. It, like other things, has its merits and demerits. Like every thing else, at first more was claimed for it than could be accomplished, and especially was this true in regard to its introduction ; at one time it was claimed that three or four hours was quite time enough to make a full set of teeth by this process; while in reality quite as much time and labor, skill and care are necessary to obtain good results as in any other style of work. This work has the advantage of a very perfect adaptation, if all the steps are properly performed. There is in this also no interstices or points of lodgment for food or foreign substances. It is lighter than any other style of work, is more congenial in the mouth, there is not that unpleasant sensation, upon the occlusion of the teeth, experienced with metal or mineral plates. The color is as yet objectionable. The character of the material is most objectionable to those who know least about it. It may be worn in the mouth with any other kind of plates. With regard to the adoption of this style of work, there are the same peculiarities observable in the profession as have already been referred to. Some test every new method for themselves. Some wait for others to make all the experiments, and determine whether a process has real merit. The remark is often made, "I got bit once with a new thing, and I'm going to be a little cautious how I take hold in future." This is a sad acknowledgment ; it is a tacit admission that the person who speaks thus, has no method of determining the merit or character of any mode or process, except by experiment. Now, while principle and theory without experiment, will not accomplish what the scientific man desires, yet simple experiment, unaccompanied by any knowledge of principles or theory, is an exceedingly feeble and uncertain guide. Every one should test every new process, at least, that promises any thing, for himself, both by experiment, and by attaining a thorough knowledge of all the principles involved, without which the man of real art and science will not rest content.

